# Distribution and Regulation of the Mobile Genetic Element-Encoded Phenol-Soluble Modulin PSM-mec in Methicillin-Resistant *Staphylococcus aureus*

**DOI:** 10.1371/journal.pone.0028781

**Published:** 2011-12-12

**Authors:** Som S. Chatterjee, Liang Chen, Hwang-Soo Joo, Gordon Y. C. Cheung, Barry N. Kreiswirth, Michael Otto

**Affiliations:** 1 Laboratory of Human Bacterial Pathogenesis, National Institute of Allergy and Infectious Diseases, The National Institutes of Health, Bethesda, Maryland, United States of America; 2 Public Health Research Institute Tuberculosis Center, University of Medicine and Dentistry of New Jersey, Newark, New Jersey, United States of America; University of Liverpool, United Kingdom

## Abstract

The phenol-soluble modulin PSM-mec is the only known staphylococcal toxin that is encoded on a mobile antibiotic resistance determinant, namely the staphylococcal cassette chromosome (SCC) element *mec* encoding resistance to methicillin. Here we show that the *psm-mec* gene is found frequently among methicillin-resistant *Staphylococcus aureus* (MRSA) strains of SCC*mec* types II, III, and VIII, and is a conserved part of the class A *mec* gene complex. Controlled expression of AgrA versus RNAIII in *agr* mutants of all 3 *psm-mec*-positive SCC*mec* types demonstrated that expression of *psm-mec*, which is highly variable, is controlled by AgrA in an RNAIII-independent manner. Furthermore, *psm-mec* isogenic deletion mutants showed only minor changes in PSMα peptide production and unchanged (or, as previously described, diminished) virulence compared to the corresponding wild-type strains in a mouse model of skin infection. This indicates that the recently reported regulatory impact of the *psm-mec* locus on MRSA virulence, which is opposite to that of the PSM-mec peptide and likely mediated by a regulatory RNA, is minor when analyzed in the original strain background. Our study gives new insight in the distribution, regulation, and role in virulence of the PSM-mec peptide and the *psm-mec* gene locus.

## Introduction

*Staphylococcus aureus* is a significant human pathogen causing a series of acute and chronic, and frequently life-threatening diseases [1]. Widespread resistance to antibiotics severely complicates management of *S. aureus* infections [2]. *S. aureus* strains that are resistant to methicillin (methicillin-resistant *S. aureus*, MRSA) in particular are widespread in the hospital setting and have recently also caused a global epidemic of community-associated infections [3]. The molecular determinants that carry methicillin resistance are located on mobile genetic elements (MGEs) called staphylococcal cassette chromosome *mec* (SCC*mec*), which range in size from 21 to 67 kb. SCC*mec* elements contain genes involved in methicillin resistance and recombination, in addition to accessory elements such as transposons, integrated plasmids, and genes encoding resistance to heavy metals [4].

The virulence potential of *S. aureus* varies significantly among different strains as a result of the presence or absence of MGEs that contain genes coding for toxins and other virulence determinants [5]. In contrast, a series of core genome-encoded toxins such as alpha-toxin are present in virtually all *S. aureus* strains. Among those, phenol-soluble modulins (PSMs) have recently emerged as crucial virulence determinants that promote pro-inflammatory processes and lyse neutrophils and other human cell types [6],[7]. In addition, some PSMs facilitate biofilm structuring and dispersal [8].

We have recently discovered a novel PSM peptide, PSM-mec, which is encoded on SCC*mec* elements, representing the first toxin gene of *S. aureus* co-localized on MGEs with antibiotic resistance determinants [9]. According to published MRSA genome sequences, the *psm-mec* gene is found predominantly within SCC*mec* elements of types II and III [9]. However, a broad-scale analysis of the distribution of *psm-mec* in SCC*mec* elements of MRSA strains has not been performed yet.

Many toxins in *S. aureus* are under control of the Agr global virulence regulator [10]. Until recently, it was believed that all Agr targets are regulated via a regulatory RNA, RNAIII [11]. However, we have shown in a recent study that selected genes are controlled by the Agr system independently of RNAIII [12]. Genes coding for PSMs are the prototypes of such genes that are controlled by the AgrA DNA-binding protein in an RNAIII-independent fashion [12]. Like other PSMs, PSM-mec is under strict control by Agr, as evidenced by complete lack of production in *agr* deletion mutants [9]. However, it has remained unclear whether the *psm-mec* gene is under RNAIII- or AgrA-dependent control.

Previously, we demonstrated that in a strain that produces high relative amounts of PSM-mec compared to other PSMs, *psm-mec* significantly increases lysis of neutrophils and erythrocytes, and increases virulence in a mouse model of skin infection [9]. Furthermore, Kaito et al. recently showed that the *psm-mec* locus also has a regulatory function, presumably via a *psm-mec*-associated regulatory RNA, whose impact on virulence is opposite to that mediated by the PSM-mec peptide [13]. This was demonstrated using plasmid-based expression of the *psm-mec* gene with or without an altered start codon. While this method allows conclusions on a regulatory function of *psm-mec*, an understanding of the role of *psm-mec* in virulence can only be gained using manipulation of the genome of the original strain background. However, the result of deleting the *psm-mec* gene in strains other than those of SCC*mec* type II has not been evaluated.

Here, we analyzed a collection of 29 MRSA strains containing the most commonly isolated SCC*mec* and sequence types (STs) to analyze the distribution and location of the *psm-mec* gene in MRSA strains in detail. Furthermore, we investigated whether the *psm-mec* gene is under control of AgrA or RNAIII and to what extent deletion of *psm-mec* impacts virulence in strains of different SCC*mec* types.

## Results

**Figure 1:**
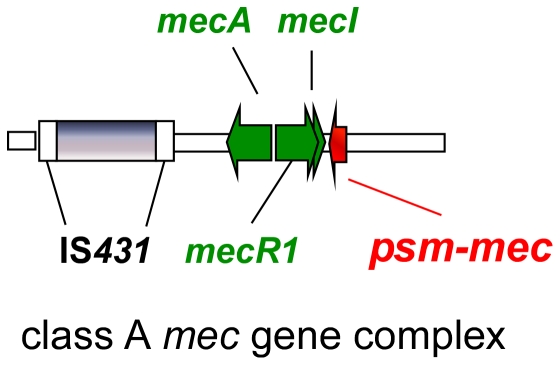
Conserved location of the *psm-mec* gene in the class A *mec* gene complex. The class A *mec* gene complex contains the core genes of the SCC*mec* element in the order IS431-*mecA*-*mecR*-*mecI*. Downstream of *mecI*, the *psm-mec* gene is encoded in opposite direction as a conserved part of this complex.

We first analyzed an MRSA strain collection containing all major SCC*mec* types for presence and location of the *psm-mec* gene. Characteristics of the analyzed MRSA strains, including clonal complex (CC), ST, *spa* type, and SCC*mec* type are shown in Table 1. We used two analytical PCR assays to determine whether the *psm-mec* gene is present and whether its location is conserved: the first PCR fragment amplified the *psm-mec* gene itself, while the second PCR tested for the connection between *psm-mec* and the adjacent *mecI* gene. Our results demonstrated presence of *psm-mec* in SCC*mec* types II, III, and VIII, i.e. those types that have a class A *mec* gene complex (containing the core genes of the SCC*mec* element in the order IS*431*-*mecA*-*mecR1*-*mecI*) [4], [14] (Fig. 1), while it was absent from types I, IV, V, and VI. Importantly, all tested type II, III, and VIII strains contained *psm-mec*, and the *psm-mec* gene was always connected to the *mecI* gene. Meanwhile, strain BK16991, possessing a truncated SCC*mec* III element (ϕSCC*mec*_16991_) that carries a class A *mec* complex but lacks the *ccr* gene complex [14], was also positive for *psm-mec* (Tab. 1). Furthermore, production of PSM-mec peptide occurred only in strains positive for *psm-mec* and the *psm-mec*/*mecI* connection (Tab. 1). This indicates that *psm-mec* and its location are conserved features of the class A *mec* gene complex.

**Table 1:** Distribution of the *psm-mec* gene and PSM-mec peptide production in MRSA strains.

Strain	Geographic origin	CC	ST	*spa* types	*spa* motif	SCCmec type	*psm-mec* gene	*psm-mec* – *mecI* link	PSM-mec peptide
NCTC10442	United Kingdom	8	250	1	YHGFMBQBLO	I			
NRS35	France	8	572	4	YHFGFMBQBLO	I			
NRS36	France	8	247	4	YHFGFMBQBLO	I			
NRS39	Scotland	8	247	40	YFGFMBQBLO	I			
Mu3	Japan	5	5	2	TJMBMDMGMK	II	**+**	**+**	
NRS74	United States	5	105	2	TJMBMDMGMK	II	**+**	**+**	
NRS382	United States	5	5	2	TJMBMDMGMK	II	**+**	**+**	**+**
NRS383	United States	30	36-like^b^	16	WGKAKAOMQQQ	II	**+**	**+**	**+**
NRS22	United States	45	45	10	A2AKEEEMBKB	II	**+**	**+**	**+**
NRS27	United States	45	45	15	A2AKEEMBKB	II	**+**	**+**	**+**
85/2082	Japan	8	239	3	WGKAOMQ	III	**+**	**+**	**+**
BK1406	United States	8	239	3	WGKAOMQ	III	**+**	**+**	**+**
BK16704	Romania	8	239	351	WGKAQQ	III^a^	**+**	**+**	**+**
BK16691	Romania	8	239	351	WGKAQQ	ϕSCC*mec*_16991_ a	**+**	**+**	**+**
NRS387	United States	5	5	29	TJMBMDMGGMK	IV			
NRS119	United States	8	507	7	YHGCMBQBLO	IV			
NRS386	United States	8	72	49	UJGFMGGM	IV			
NRS385	United States	8	8	7	YHGCMBQBLO	IV			
NRS271	United Kingdom	22	22	382	TJJEJNF2MNF2MOMOKR	IV			
NRS241	United States	59	59	17	ZDMDMNKB	IV			
NRS255	France	80	80	70	UJGBBPB	IV			
BK19489	United States	88	78	868	UFKBBPB	IV			
NRS265	Switzerland	88	88	876	UGFMEBBPB	IV			
BK23603	United States	8	8	664	YC2BQBLO	V			
WIS	Australia	45	45	6	A2AKBEKBKB	V			
HTO826	France	152	377	207	UJ2GMKKPNSG	V			
HDE288	Portugal	5	5	45	TJMBDMGMK	VI			
BK20781	United States	8	8	1	YHGFMBQBLO	VIII	**+**	**+**	**+**
BK23684	Canada	8	8	544	YHGFC2BQBLO	VIII	**+**	**+**	**+**

^*a*^ϕSCC*mec*_16991_ is a truncated SCC*mec* III element, with a 24-kb deletion encompassing the right chromosomal junction downstream of the class A *mec* element; BK16704 possesses an SCC*mec* 3A.1.4 structure, with a *dcs* gene locus located at the J3 region of SCC*mec* element [19].

^*b*^single locus variant of ST36, with the MLST allelic profile 2-2-52-2-3-3-2.

**Figure 2:**
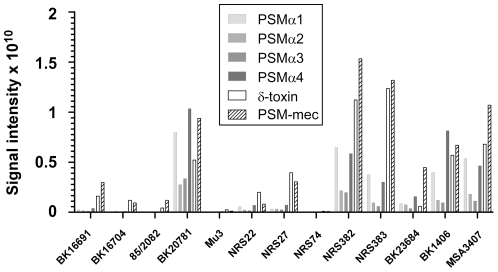
PSM production in *psm-mec*-positive MRSA strains. All MRSA strains that were determined to be *psm-mec*-positive in this study were analyzed for production of all *S. aureus* PSM peptides (α1, α2, α3, α4, β1, β2, δ-toxin, mec) by RP-HPLC/MS. Production levels are shown for all peptides except for β1 and β2, for which production levels were in general very low. The previously analyzed MRSA strain MSA3407 is shown as reference.

Expression of *psm* genes can vary considerably, with functionality of the Agr system having a strong effect on PSM production [7], [15]. Analysis of the presence of the *psm-mec* gene alone therefore only allows a preliminary assessment of the potential of a given strain to produce the PSM-mec peptide. Furthermore, our previous results indicated that the extent to which PSM-mec contributes to virulence is influenced to a large extent by the relative level of production compared to that of other PSMs [9]. Therefore, we first determined concentrations of all PSM peptides in culture filtrates of every MRSA strain of our collection that was positive for the *psm-mec* gene (Fig. 2). Absence of PSM-mec production in *psm-mec*-positive strains occurred in two strains (Mu3 and NRS74). Both these strains lacked production of any PSM peptide, indicating that this phenotype is due to Agr being non-functional. In strains with production of PSM-mec, concentrations relative to those of other PSMs varied. Strains BK16691 and BK23684 for example showed moderate absolute, but high relative production of PSM-mec compared to other PSMs, similar to strain MSA890 (SCC*mec* type II), in which PSM-mec has been shown in a mouse skin infection model to contribute significantly to virulence [9].

PSM-mec concentration was strongly correlated with concentrations of AgrA-controlled PSMα peptides (PSMα1, p = 0.0002; PSMα2, p = 0.0008; PSMα3, p = 0.014; PSMα4, p = 0.0048; Pearson's correlation test) and the RNAIII-embedded δ-toxin (p<0.0001). Correlation with production of PSMβ peptides was not or barely significant, likely owing to the low level of PSMβ peptide concentration under the investigated conditions. Finally, there was no apparent correlation between SCC*mec* type and level of PSM production.

**Figure 3:**
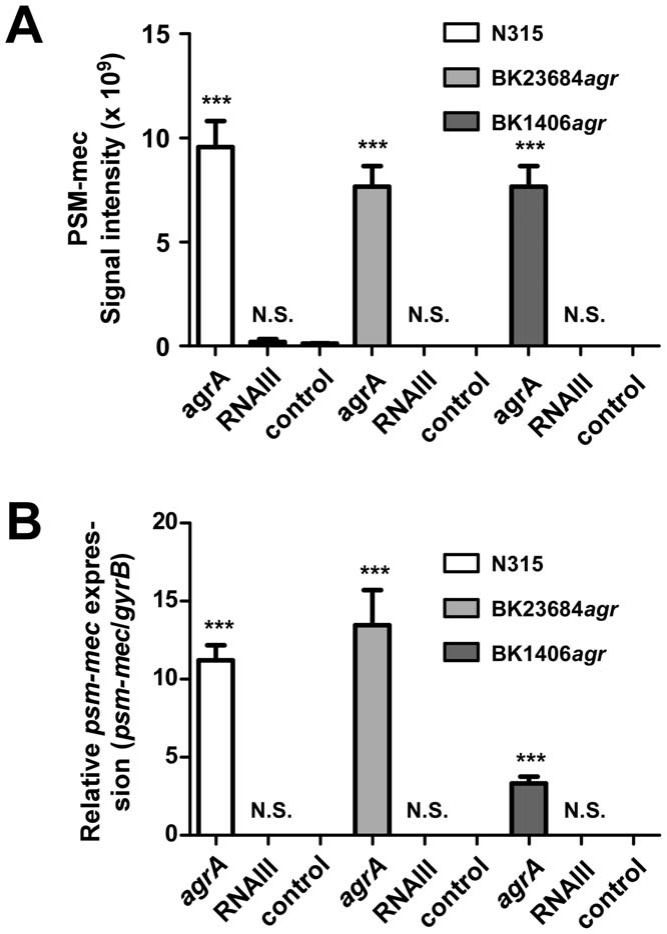
Mechanism of Agr-dependent regulation of *psm-mec*. Expression of *psm-mec* on the protein (A) and transcript (B) levels was analyzed in the *psm-mec*-positive, Agr-dysfunctional MRSA strains N315 (SCC*mec* type II), BK1406*agr* (SCC*mec* type III), and BK23684*agr* (SCC*mec* type VIII), in which the *agrA* gene or RNAIII were constitutively over-expressed using plasmid pTXΔ (in strain N315) or expressed using induction with 0.5% xylose in a pKX background (in the other 2 strains). Control, containing the empty plasmid pTXΔ16 or pKX16, respectively. (A) Protein level analysis. (B) Transcript level analysis. Relative expression of the *psm-mec* transcript is shown, relative to expression of the housekeeping gene *gyrB*. N.S., not significant; ***, p<0.0001; One-way analysis of variance with Dunnett post tests compared to the control sample.

As the correlation analysis did not allow a clear distinction of whether expression of the *psm-mec* gene is under control of AgrA or RNAIII, we constitutively expressed *agrA* or RNAIII from plasmids in an *agr*-negative strain background, using the naturally *agr*-dysfunctional, *psm-mec*-positive MRSA strain N315 [9], [16]. Furthermore, we produced *agr* mutations in strain BK23684 (SCC*mec* type VIII,) and strain BK1406 (SCC*mec* type III). We then determined expression of *psm-mec* on the transcript and protein levels. These analyses clearly showed that *psm-mec* is under transcriptional control by the DNA-binding AgrA response regulator in all 3 strains, while RNAIII had no influence on *psm-mec* expression (Fig. 3A,B). Despite its unusual location on an MGE, the *psm-mec* gene is thus regulated in the same fashion as the other *psm* operons, *psm*α and *psm*β [12]. Interestingly, the putative *psm-mec* promoter region does not contain sequences with strong homology to those of the *psm*α, *psm*β, or *agr* P2 and P3 promoter regions. This finding is in keeping with our previous analysis of those promoters [12], which showed only little conservation of AgrA binding consensus sequences, underlining that AgrA-regulated genes cannot be identified based on sequence analysis alone.

**Figure 4:**
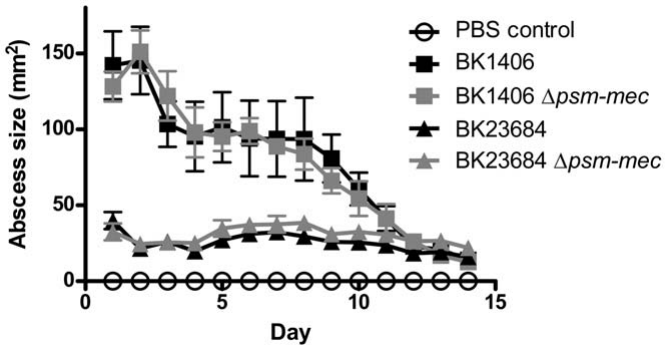
Mouse abscess model. Isogenic *psm-mec* deletion mutants of strains BK1406 (SCC*mec* type III) and BK23684 (SCC*mec* type VIII) were compared to their corresponding wild-type strains in a mouse abscess model that was performed as previously described for strain MSA890 and its *psm-mec* deletion mutant [9]. Abscesses formed by strain BK1406 usually presented with open lesions, whereas those formed by strain BK23684 did not. There were no significant differences (using t-tests) between corresponding wild-type and *psm-mec* deletion mutant strain abscess sizes.

**Figure 5:**
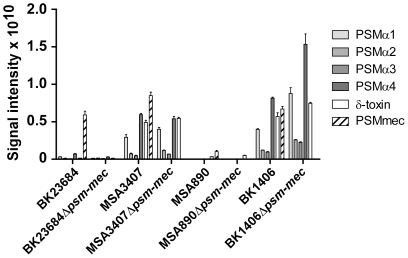
Impact of the *psm-mec* locus on the production of genome-encoded PSMs. Four MRSA strains of 3 different SCC*mec* types and their isogenic *psm-mec* deletion mutants were analyzed for production of all *S. aureus* PSM peptides (α1, α2, α3, α4, β1, β2, δ-toxin, mec) by RP-HPLC/MS. Production levels are shown for all peptides except for β1 and β2, for which production levels were in general very low.

Recently, Kaito et al. demonstrated a regulatory effect of the *psm-mec* RNA when over-expressed on a plasmid in hosts without SCC*mec* or with SCC*mec* type IV not harboring *psm-mec* [13]. Furthermore, under those circumstances the *psm-mec* transcript down-regulated expression of the cytolytic PSMα3 peptide and led to decreased virulence at least in part for that reason [13]. Of note, this report replaced, or added to, a previous hypothesis by the same authors, attributing that regulatory effect to a putative gene named *fudoh* [17], which overlaps with almost the entire *psm-mec* gene. Given the clear evidence for *psm-mec* expression [9], “*fudoh*” very likely is a pseudogene. In contrast, in our previous study, deletion of *psm-mec* in an SCC*mec* type II strain with high relative production of PSM-mec compared to other PSMs (MSA890) resulted in increased virulence and hemolysis, while in an SCC*mec* type II strain with lower relative production of PSM-mec (Sanger 252), virulence was not significantly changed [9]. Furthermore, we demonstrated a function of the PSM-mec peptide in cytolysis, indicating an important role in promoting disease [9]. To gain further insight into the role of the *psm-mec* locus in its different original strain backgrounds, we here analyzed two strains of SCC*mec* types not previously included in our analyses: one strain each of SCC*mec* type III and VIII (BK1406, BK23684), with moderate to high relative production of PSM-mec. We did not detect a significant change in virulence upon deletion of the *psm-mec* locus (Fig. 4). Additionally, we investigated in those 4 *psm-mec* deletion strains, containing examples of all SCC*mec* types in which *psm-mec* occurs, whether the genomic deletion of the *psm-mec* locus led to a change in the production of PSM peptides. We found only minor and inconsistent changes in PSM production of *psm-mec* deletion versus the corresponding wild-type strains (Fig. 5).

## Discussion

The staphylococcal PSM-mec toxin is unique in its link to an antibiotic resistance element. This coupling allows simultaneous acquisition by *S. aureus* strains of key antibiotic resistance together with virulence determinants, significantly facilitating the spread of antibiotic resistance and virulence among the *S. aureus* population [9], [18]. The present study was undertaken because, despite the immense importance of such a mechanism for public health, we lack knowledge about distribution and location of the *psm-mec* gene.

Results from the present study show that the MGE-encoded *psm-mec* gene is widely distributed among MRSA strains and its expression is highly variable. The *psm-mec* gene is linked to the class A *mec* gene complex present in SCC*mec* types II, III, and VIII, with a conserved location next to the *mecI* gene. The fact that the *psm-mec* gene was always found among strains harboring SCC*mec* elements of types II, III, and VIII at the same location indicates that it is an original part of these elements, representing a possible marker for analytical purposes.

RNAIII-independent regulation via the Agr system has only recently been discovered, and the *psm*α and *psm*β operons are the only genetic elements for which a direct binding of and regulation by the AgrA response regulator has been shown on a molecular level [12]. These findings indicated that PSMs represent evolutionarily early parts of the Agr regulon, while other targets of Agr regulation likely were added later, via evolution of the regulatory RNAIII and its connection to the Agr system. This raised the interesting question whether *psm-mec* is under the same, direct regulation of AgrA despite its location on an MGE. We demonstrate here that *psm-mec* shares with the other, core-genome encoded *psm* genes the characteristic Agr-dependent, but RNAIII-independent regulation. Thus, based on this similarity, it is tempting to speculate that core genome-encoded *psm* genes and *psm-mec* have a common origin.

Finally, we analyzed the contribution of *psm-mec* to virulence in SCC*mec* type III and VIII strains. We do not believe that the type of SCC*mec* element impacts the contribution of *psm-mec* to virulence, as the region around *psm-mec* is well conserved in SCC*mec* elements of class A. Rather, these experiments were performed to gain insight into the role of the *psm-mec* locus in additional strains. Our previous results on the cytolytic role of the PSM-mec toxin [9] and those by Kaito et al. [13] who found a regulatory effect of the *psm-mec* locus, including on genome-encoded PSMs, indicate that the contribution to virulence of the RNA and the peptide it encodes are opposite. However, the results by Kaito et al. were based entirely on plasmid-based over-expression in strains not naturally harboring *psm-mec*, prompting us to investigate the role of the *psm-mec* locus deletion by allelic replacement strategies, which are not influenced by plasmid copy effects and allow conclusions on the role of the locus in its natural strain background. In the 4 clinical strains that we investigated in our previous and the present study, absence of *psm-mec* either did not significantly alter virulence in a mouse skin infection model, or virulence was decreased. Furthermore, there were only very minor effects of the *psm-mec* deletion on the expression of other PSM peptides. Notably, this included the strongly cytolytic PSMα3, whose production was reported as decreased upon plasmid-based expression of *psm-mec* by Kaito et al. [13]. These findings suggest that the virulence-promoting effect of the PSM-mec peptide balances or supersedes the reported virulence-diminishing effect of the *psm-mec* RNA, depending on the strain background. In the future, a thorough analysis of the roles of *psm-mec* on the RNA and peptide levels will have to be accomplished using manipulations of *psm-mec*-containing clinical strains on the genome that allow a distinction between the roles of the *psm-mec* transcript and its product.

## Methods

### Ethics statement

Animal studies were carried out in strict accordance with the recommendations in the Guide for the Care and Use of Laboratory Animals of the National Institutes of Health. The protocol was approved by the Animal Care and Use Committee (IUCAC number ASP LHBP 1E), National Institute of Allergy and Infectious Diseases.

### Bacterial strains, typing, and growth conditions

The bacterial strains used in the present study are shown in Table 1. In addition, we used strains N315 and MSA3407, which were analyzed in our previous study on *psm-mec* [9]. SCC*mec* and *spa* typing were performed as previously described [14], [19]. All bacteria were grown in tryptic soy broth (TSB). For xylose induction experiments, glucose-free TSB was used. Antibiotics were added at appropriate concentrations (chloramphenicol, 10 µg/ml, tetracycline 12.5 µg/ml).

### Production of *psm-mec* and *agr* mutants

Isogenic deletion mutants of the *psm-mec* locus in strains BK1406 and BK23684 were produced using allelic replacement with plasmid pKOR1 as described [9], [20]. Mutations in *agr* were produced by phage transduction from strain RN6911 (in strain BK23684), or by selection for a spontaneous *agr* mutation using a hemolysis screen for strain BK1406. The latter was necessary as a directed mutagenesis procedure via allelic replacement was not possible in any of the SCC*mec* type III strains in our collection, as they all carried a series of antibiotic resistances. The mutation in BK1406*agr* was mapped to base 739 (C to A), causing an amino acid substitution at the 247^th^ position of AgrC (proline to threonine). Mutants in the *agr* locus were complemented with plasmids pTXΔRNAIII, pTXΔ*agrA* [12] or a newly constructed pKX*agrA*. The plasmid pKX*agrA* contains kanamycin instead of tetracycline resistance and was constructed using the same primers as pTXΔ*agrA* (for all oligonucleotides, see Table 2).

**Table 2:** Oligonucleotides used in this study.

Name	Sequence
*For psm-mec analytical PCR*	
Psmmec-for	ATGGATTTCACTGGTGTTATTACAAGC
Psmmec-rev	TTAACCGAAAGCCTGAATGCAAGTCTTG
*For mecI link analytical PCR*	
Psmmec-for	ATGGATTTCACTGGTGTTATTACAAGC
mecI-rev	TACAAATGCAAAAGGACTGGAGTCC
*For RT-PCR*	
psmmecRTfor	TGCATATGGATTTCACTGGTGTTA
psmmecRTrev	ATTTAATCAAGACTTGCATTCAG
psmmecRTprobe	CGTTGAATATTTCCTCTGTTTTTTAGTTG

### Analysis of PSM-mec production

Production of PSM-mec was analyzed by reversed-phase high pressure liquid chromatography/electrospray mass spectrometry (RP-HPLC/ESI-MS) of culture filtrates as described [7], [9].

### Quantitative real-time PCR

Quantitative real-time PCR (qRT-PCR) was performed as described [21], using the house-keeping gene *gyrB* as control.

### Animal skin infection model

The mouse skin infection (abscess) model was performed as described previously [22].

### Statistics

Statistical analysis was performed using graph Pad Prism Version 5.04.
